# Inferring Cuisine - Drug Interactions Using the Linked Data Approach

**DOI:** 10.1038/srep09346

**Published:** 2015-03-20

**Authors:** Milos Jovanovik, Aleksandra Bogojeska, Dimitar Trajanov, Ljupco Kocarev

**Affiliations:** 1 Faculty of Computer Science and Engineering, Rugjer Boshkovikj 16, 1000, Skopje, Macedonia

## Abstract

Food - drug interactions are well studied, however much less is known about cuisine - drug interactions. Non-native cuisines are becoming increasingly more popular as they are available in (almost) all regions in the world. Here we address the problem of how known negative food - drug interactions are spread in different cuisines. We show that different drug categories have different distribution of the negative effects in different parts of the world. The effects certain ingredients have on different drug categories and in different cuisines are also analyzed. This analysis is aimed towards stressing out the importance of cuisine - drug interactions for patients which are being administered drugs with known negative food interactions. A patient being under a treatment with one such drug should be advised not only about the possible negative food - drug interactions, but also about the cuisines that could be avoided from the patient's diet.

The task of identifying and obtaining foods which contribute to the overall human health, satisfy nutritional and energy needs and at the same time not inducing food poisoning, has been around as long as humans have. Human diet, which provides essential nutrients for the organism health, is influenced by different factors, including cultural habits, socio-economic status, and weather/climate. For example, spice consumption in the warm regions is tightly connected to the need for keeping the food resistant to bacteria for a longer period of time^1^.

It is a well known fact that certain foods can influence the effect of a drug in an organism^2^,^3^,^4^,^5^. The food-induced changes in the bioavailability (the degree and rate at which a drug is absorbed into someone's system) of the drug modify the clinical effect of the drug. Generally, food - drug interactions can result in significant reduction of the drug bioavailability, either by direct interaction between a substance from the food and a chemical component of a drug, or by the physiological response of food intake (e.g. gastric acid secretion). This can often result in treatment failure. Additionally, food - drug interactions can result in an increase in drug bioavailability, either by increased drug solubility as a direct result from a substance from the food, or indirectly, by food-initiated secretion of gastric acid or bile. Even though this leads to an increase in the effect of the drug, it can often result in serious toxicity^2^.

The highest selling drugs in the world include antineoplastic and immunomodulating agents, respiratory system drugs, alimentary tract and metabolism drugs, cardiovascular system drugs, and nervous system drugs^6^. Recent statistics report that nearly 70% of the American population consumes at least one prescription drug, a number which was only 48% in 2010. Twenty percent of them are on five or more drugs^7^. According to Ref. 8, an alarming number of 1.5 million people are harmed by medications, including the errors due to the lack of information provided by the pharmacist or the unawareness of the patient to read and follow the patient drug information.

Different world regions use diverse ingredients and foods as part of their cuisines and therefore the negative food-induced interactions with drugs vary from one part of the world to another. In addition, as non-native cuisines are becoming increasingly popular (due to travel and due to their appearance in almost every corner of the planet as a result of global cultural exchange), the effect different cuisines have on certain drugs or categories of drugs is becoming very important.

Although food - drug interactions are well studied, much less is known about cuisine - drug interactions. The food - drug interactions are clinically proven interactions between a drug and a given ingredient (ex. milk, avocado, garlic), and with cuisine - drug interactions we define the interactions between a drug and a world cuisine, using information extracted from the food - drug interactions and the usage of the interacting ingredients in a specific cuisine. The aim of the analysis presented here is to address the distribution of the known negative food - drug interactions in the world cuisines. The influence that world cuisines have on different drug categories is analysed by transforming and connecting two datasets (drug data and recipes data) into a novel structure empowered with the concept of Linked Data^9^. The results are striking: North American cuisine has the most negative interactions (10.242‰) with drugs from the category ‘Antiinfectives for systemic use'. In other words, 10 out of 1,000 patients could possibly have negative effects when being administered this category of drugs. Similarly, European cuisines (from Western Europe, Northern Europe and Eastern Europe) have the most negative interactions with drugs from the same category (‘Antiinfectives for systemic use'). On the other hand, the cuisines from Southern Europe, Asia, Latin America and Africa negatively interact mostly with drugs from the categories ‘Blood and blood forming organs' and ‘Various'.

Our main message from this work could be summarized as guidance in the form: “if you are being administered drugs from a certain drug category, be aware of what cuisines you should be consuming”.

## Results

Two different aspects of food - drug interactions are considered: (1) negative interactions between drugs from a given category and recipes from a given cuisine, and (2) ingredients' impact on the negative food - drug interactions in different parts of the world.

### Cuisine - drug category interactions

For the analysis of negative interactions between a particular drug category, i.e. a category of drugs grouped by their Anatomical Therapeutic Chemical (ATC) classification codes (Table 6), and a cuisine, we calculate the permils of existing interactions between them (Table 1) using Equation 1.

This ratio represents the probability of a negative food interaction to occur when a patient using a drug from a given category consumes a meal from a given cuisine. We use the measurement of permils for the ratio in order to show the number of patients, out of 1,000, which can have a negative food interaction when combining the drug category with the cuisine. The aim of this analysis aspect is to identify the negative impact of consuming foods from specific cuisine while taking a prescribed drug.

The results (Table 1) show that some of the most intensive negative food - drug interaction occur between drugs from category B and recipes from Asian, African and Latin American cuisines, drugs from category J and recipes from North America and Europe, as well as drugs from category V and recipes from almost the entire world. Also, we can note that drugs from categories H, P and R have a very rare occurrence of negative interactions with food, and drugs from category M have no interactions at all.

The results from Table 1 help us distinguish three different patterns of food - drug interactions from cuisine and drug category points of view.

#### Pattern 1

**Figure 1:**
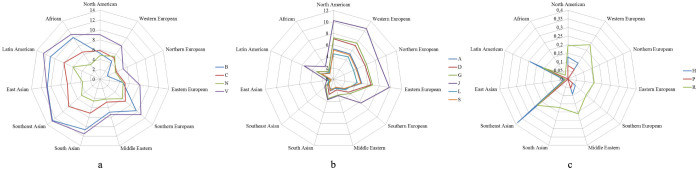
Negative food - drug interactions between drugs from a category and foods from a cuisine, expressed in permils. Figure (a) depicts the interactions of drugs from categories B, C, N and V. The drugs from these four categories have more negative food - drug interactions with recipes from South Europe, the Middle East, South Asia, Southeast Asia, East Asia, Latin America and Africa, as opposed to other cuisines. Figure (b) depicts the interactions of drugs from categories A, D, G, J, L and S. These drugs have more intensive negative food - drug interactions with recipes from North America, Western Europe, Northern Europe and Eastern Europe, as opposed to other cuisines. Figure (c) depicts the interactions of drugs from categories H, P and R. These drugs have a significantly smaller ratio of interactions compared to those from (a) and (b).

The first pattern consists of drugs from categories B, C, N and V. As shown in Table 1, the drugs from these four categories have more negative food - drug interactions with recipes from South Europe, Middle East, South Asia, Southeast Asia, East Asia, Latin America and Africa, as opposed to other cuisines. This pattern is depicted on Fig. 1a.

The reason behind this pattern of influence is the fact that the drugs from these four categories have negative food interactions with garlic and ginger. These two ingredients are largely present and directly responsible for the negative food - drug interactions in these geographical regions (Table 3, Table 4). Additionally, the negative food interactions these drugs have with avocado, licorice and grapefruit, add up to the difference between these drug categories and the rest. The interactions with coffee are also present within these categories, but they are present in other categories as well, so the effect of coffee is not much evident in the specifics of this pattern.

**Figure 2:**
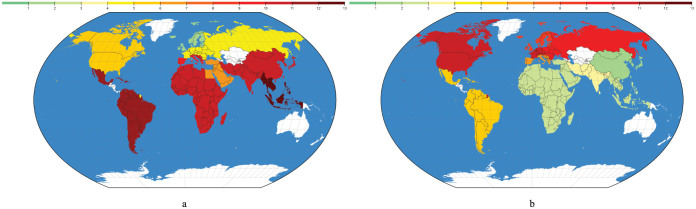
Number of patients (per 1,000) with possible negative food - drug interactions while being administered a category B or category J drug, in different cuisines, globally. Figure (a) depicts the global distribution of negative interactions involving a category B drug. These drugs have significantly more interactions with recipes from South Europe, the Middle East, South Asia, Southeast Asia, East Asia, Latin America and Africa. Figure (b) depicts the global distribution of negative interactions involving a category J drug. These drugs have significantly more interactions with recipes from North America, Western Europe, Northern Europe and Eastern Europe. The maps were generated using the d3.js library (http://d3js.org).

Fig. 2a depicts the intensity of negative food - drug interactions for drugs from the B category, shown in a color scale. The figure shows the number of patients out of 1,000, which are being administered a drug from category B, which can have a negative interaction with a recipe from a cuisine. The white areas on the map represent the cuisines we don't have any data on.

Since category B drugs belong to the first pattern, Fig. 2a shows the same negative food - drug interaction intensity we see in Fig. 1a, i.e. drugs from category B have significantly more interactions with recipes from South Europe, Middle East, South Asia, Southeast Asia, East Asia, Latin America and Africa.

#### Pattern 2

The second pattern consists of drugs from categories A, D, G, J, L and S. These drugs have a significant ratio of negative food - drug interactions with recipes from North America, Western Europe, Northern Europe and Eastern Europe, as opposed to other cuisines. This pattern is depicted on Fig. 1b.

The emergence of this pattern is mainly due to the negative food interactions which drugs from these categories have with milk. As we can see from Table 2, milk is the number one cause for negative food - drug interactions globally, and Table 3 and Table 4 show that it is the primary source of negative interaction in these regions of the world. Since these cuisines use milk in a large portion of their recipes, unlike the rest of the cuisines, this pattern is expected.

Fig. 2b depicts the intensity of negative food - drug interactions for drugs from the J category. Since category J drugs belong to the second pattern, this figure shows the same negative food - drug interaction intensity we see in Fig. 1b, i.e. drugs from category J have significantly more interactions with recipes from North America, Western Europe, Northern Europe and Eastern Europe.

#### Pattern 3

The remaining drug categories, H, P and R, have significantly smaller negative food - drug interactions ratio compared to other categories. They form a different pattern, depicted on Fig. 1c, but due to the very small ratio of interactions, this pattern is not as compact as the other two. The drugs from these categories have negative effects when interacting with coffee, tea and grapefruit (Table 5), and not with other ingredients. As we see from Table 3 and Table 4, coffee is a top three interacting ingredient in North America, Europe, the Middle East and in Latin America, which corresponds with the pattern on Fig. 1c. The high use of grapefruit and tea in recipes from Southeast Asia (Table 4) is responsible for the high interactions of drugs from these categories with this cuisine.

### Ingredient analysis

The second aspect of the analysis aims to detect the main ingredients involved in the negative food - drug interactions. Table 2 shows the percentage of negative food - drug interactions in which an ingredient is involved and is responsible for the negative interaction. The percentage is calculated out of the total number of negative food - drug interactions we discovered in the analysis, which is 298,762 interactions.

Table 2 clearly shows that two ingredients are most common in the negative interactions: milk, responsible for with over 56% of the negative interactions, and garlic with over 22%.

These two ingredients have different effects on the food - drug interactions in the parts of the world, and within distinct drug categories. Table 3 shows the top three ingredients per cuisine which are involved in the negative interactions. As we can see, milk is the ingredient which causes most of the negative interaction in North America, Western, Northern and Eastern Europe. On the other hand, the most problematic ingredient in recipes from South Europe, Middle East, Asia, Latin America and Africa, is garlic.

**Figure 3:**
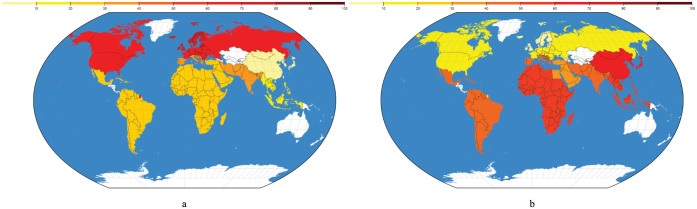
Percentage of occurrence of milk and garlic in negative food - drug interactions in different cuisines, globally. Figure (a) depicts the percentage of occurrence of milk, which causes most of the negative interaction in North America, Western, Northern and Eastern Europe. Figure (b) depicts the percentage of occurance of garlic, which is the most problematic ingredient in recipes from South Europe, the Middle East, Asia, Latin America and Africa. The maps were generated using the d3.js library (http://d3js.org).

Fig. 3 shows this trend on a world map. Fig. 3a illustrates the percentage of occurrences of milk in the total number of negative food - drug interaction within a cuisine, while Fig. 3b depicts the same impact of garlic.

A more complete overview of the impact some ingredients have in different parts of the world is presented in Table 4. We see here that the most common ingredients responsible for the negative food - drug interactions are milk, garlic, coffee and ginger, while the other ingredients have significantly lower impact. However, out of these four ingredients, milk and garlic have the most notable impact (Table 2, Table 3, Table 4), so the discussion will be focused on them.

#### The impact of milk

Milk has been proved to have negative effect generally on antibiotics^2^,^5^, and antibiotics belong to categories A, C, D, G, J, L and S^10^. Milk reduces the bioavailability and even prevents the absorption of some of these drugs. This corresponds with our results in Table 5.

The reasons for such high occurrence of milk in negative food - drug interactions in the western culture (Fig. 3a, Table 3, Table 4) may be a result of the high use of milk and dairy products in general in this part of the world. The consumption of milk in the western culture, and especially in Northern Europe where the percentage of occurrence of milk in the interactions is the largest, is probably a direct consequence of the high lactose tolerance the population from these regions has. Countries from Europe, and especially Northern Europe, are known to have the highest percentage of lactose tolerant population in the world^11^,^12^,^13^. On the other hand, regions such as Southeast Asia, East Asia and South Africa, are known as regions with high percentage of lactose intolerant population^12^. This probably has a direct cause on the consumption levels of milk in those regions, leading to a decrease in the negative effects milk has in food - drug interactions in this parts of the world.

#### The impact of garlic

The reason garlic is part of more than 22% of all negative food - drug interactions we detect (Table 2), is its negative interactions with anticoagulant drugs^14^,^15^, which belong to categories B, C and S. Table 3 and Table 4 clearly show that garlic is largely responsible for the negative food - drug interactions in South Europe, the Middle East, Asia, Latin America and Africa. Its impact is evident in the rest of the world, but with much less intensity. This pattern of garlic use in various cuisines probably has some cultural and historical background, with garlic being used in Egypt, Greece, Rome, China and India since ancient times, for prevention and treatment of disease, for providing strength and increasing work capacity of laborers, and even as a performance enhancing agent for Olympic athletes^16^.

## Discussion

By transforming and connecting two datasets (drug data and recipes data) we have generated an open semantic structure (dataset), available on the Web, which has provided a basis for a cuisine - drug category interactions analysis, showing how drugs from different categories interact with recipes from different cuisines. Two patterns of negative interactions could be stressed: drugs from categories B, C, N and V have a negative impact on foods from South Europe, Asia, Latin America and Africa, whereas drugs from categories A, D, G, J, L and S negatively interact with foods from North America and Europe (Western Europe, Northern Europe and Eastern Europe). These patterns arise from the diverse ingredients used in the world cuisines, with milk being mostly responsible for the first pattern, and garlic and ginger for the second. The impact of the milk and garlic varies in different parts of the world, mainly because of the cultural, historical and biological reasons of their presence (or lack thereof) in the recipes in a given cuisine.

Our work is aimed towards stressing out the importance of professional guidance of patients which are on drugs with known negative food interactions. A patient being under a treatment with one such drug should be advised by a pharmacist or a doctor about the foods, the ingredients and the cuisines that should be avoided (or even excluded) from his/her diet. Our analysis of the global distribution of negative interactions between different drugs and cuisines can provide a general overview and a general guide for the dangers of making bad cuisine - drug combinations.

## Methods

In order for the analysis to be relevant, we acquire real-world data about drugs and recipes. Various factors influenced our decision on which sources to use to obtain the data: the validity of the data, its volume and how up-to-date they were.

Here we describe the process of choosing, obtaining and transforming the two datasets used in the analysis. In order to bring the datasets to a common representational model, we transform and publish them into datasets which follow the Linked Data principles^9^.

### The drugs dataset

Over the past years, various databases with drug data have become freely available on the Web. The Semantic Web Health Care and Life Sciences (HCLS) Interest Group (http://www.w3.org/blog/hcls/), a W3C group focused on using Semantic Web technologies in the fields of health care, life sciences, clinical research and translational medicine, has been working on transforming a range of health data from the Web into a comperhensive dataset based on Semantic Web technologies and Linked Data principles. As a result of this work, the HCLS Interest Group has deployed the Linked Open Drug Data (LODD) Cloud^17^, which contains over 380 million RDF triples (http://www.w3.org/wiki/HCLSIG/LODD/Data).

Part of the LODD Cloud is the DrugBank dataset, a Semantic Web version of the DrugBank database. The DrugBank database is a free database which provides chemical, pharmacological and pharmaceutical data for over 6,800 drugs. Since its release in 2006, the DrugBank database has been widely used for research and educational purposes by pharmacists, medical chemists, pharmaceutical researchers, clinicians, educators and the general public^18^. Because of this, it has been selected by the HCLS and included into their efforts. The DrugBank dataset in the LODD Cloud holds the same data for over 4,700 drugs in RDF format and provides a SPARQL endpoint (http://wifo5-03.informatik.uni-mannheim.de/drugbank/) for remote data access.

Among other data, the DrugBank dataset contains information about the food interactions of the drugs. There are 968 food - drug interactions in the dataset, which connect 525 different drugs with various food indications. These indications contain a reference to one or more ingredients and are mostly negative, such as “Avoid alcohol.” or “Do not take with milk.” However, there are cases where the food interaction is neutral (“Take without regard to meals.”) as well as cases where the interaction is actually positive (“Increase dietary intake of magnesium, folate, vitamin B6, B12, and/or consider taking a multivitamin.”)

Therefore, we need to precisely denote the sentiment of each food - drug interaction. In order to achieve this, we obtain a local copy of the parts of the DrugBank dataset which we need for the analysis and create new RDF properties for the dataset, which denote the different types of food interactions: negative, neutral and positive. We do the sentiment analysis of the food interactions of all drugs from the drugs dataset in a semi-automatic fashion. Then, for each drug from the dataset we analyze the negative food interactions, detect the ingredients mentioned, and locate those ingredients in the recipes from the recipes dataset. For each such drug - recipe pair we add a new relation in the drugs dataset, denoting that the drug has a negative food interaction with the recipe. This interlinking of datasets is enabled by the use of Linked Data principles and the RDF data model. As a tool for storing, interlinking and querying the datasets we use a Virtuoso Universal Server instance (http://linkeddata.finki.ukim.mk/). Using the SPARQL query language over the SPARQL endpoint of the Virtuoso instance, we are then able to query both datasets and extract the necessary data needed for the analysis.

Our extended version of the DrugBank dataset is published following the Linked Data principles and is available via a public SPARQL endpoint (http://linkeddata.finki.ukim.mk/sparql/).

### The recipes dataset

With the expansion of the Web and the presence of mobile and web applications in everyday life, there is a significant increase of the availability of online recipes and recipe datasets, which provide easy and quick access to millions of recipes from various cuisines around the world. The general intent of these datasets is merely to provide the users with everyday ideas for preparing meals and with useful information for ingredients combinations. Some of them provide even personalized ingredient shopping lists, menu planers, user ratings, etc.

Some of the recipes are available as commercial datasets and are intended for usage from mobile applications: Yammly (https://developer.yummly.com/), Food2Fork (http://food2fork.com/about/api), BigOven (http://api.bigoven.com/), and others are available on websites and are free for use: Allrecipes.com (http://allrecipes.com), Epicurious (http://www.epicurious.com/), Taste.com.au (http://www.taste.com.au/), Foodnetwork.com (http://www.foodnetwork.com/), etc.

For the purposes of this work we use the available recipes dataset provided in Ref. 19. The dataset is created using recipes from three different sources: *allrecipes.com*, *epicurious.com*, and *menupan.com*. The data file contains information for a total of 56,458 recipes, their ingredients and the cuisine of origin. The recipes are divided in 11 cuisines: North American (41,525), Western European (2,659), Eastern European (381), Southern European (4,180), Northern European (250), Middle Eastern (645), South Asian (621), Southeast Asian (457), East Asian (2,512), Latin American (2,917), African (352). In order to enable interoperability between the datasets, we transform the cleaned data from the CSV file into an RDF dataset. For the CSV-to-RDF transformation we use the Food Ontology (http://data.lirmm.fr/ontologies/food), which allows us to denote the cuisine and ingredients for all the recipes from the dataset. We do not extend the dataset further, since we already create new relations in the drugs dataset pointing from a drug to a specific recipe in the recipes dataset. We then publish the recipes dataset in Linked Data format, in the same manner as the drugs dataset. The recipes dataset is publicly available via the same SPARQL endpoint, as well.

### Analysis

After collecting and refining the two datasets, we load them into a Virtuoso Universal Server instance which is publicly available (http://linkeddata.finki.ukim.mk/) and provides endpoint-based web service access to the data from both datasets, in RDF.

The analysis is done by using the SPARQL endpoint for querying our drugs and recipes datasets. We use SPARQL queries which make use of the relations in the drugs dataset which connect the recipes a drug has negative food interactions with, and analyze various aspects of the domain and the results which arise.

The measurement of permils used to assess the ratio of interactions between a drug category and cuisine is given with:
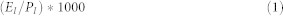
where *E_I_* is the number of existing interactions found from the datasets, and *P_I_* is the number of possible interactions between the drug category and the cuisine, calculated as the number of drugs in the category multiplied by the number of recipes in the cuisine. We use the measurement of permils for the ratio in order to show the number of patients, out of 1,000 patients treated with a drug from a given category, which can have a negative food interaction when consuming a meal from the cuisine. To calculate *E_I_*, the number of existing interactions between a drug category and a cuisine, we count the existing negative food interactions a drug from a given drug category has with recipes from a given cuisine.

To illustrate this analysis, we can use a specific example: the interaction between Oxazepam and tea. Oxazepam is a benzodiazepine used for the treatment of anxiety disorders, alcohol withdrawal, and insomnia. According to DrugBank, it has three clinically proven food - drug interactions: (a) avoid alcohol, (b) avoid excessive quantities of coffee or tea (caffeine) and (c) take with food. In our analysis, we conclude that (a) and (b) are negative food interaction of Oxazepam with alcohol, coffee, tea and caffeine. The (c) interaction is considered a positive food interaction of Oxazepam and is not taken into account for our analysis. Oxazepam is a drug categorized in the ATC category N; its ATC code is N05BA04.

On the other hand, tea is an ingredient in 102 distinct recipes from 8 cuisines in our dataset. One of the 102 recipes is recipe #9966, “Kumquat-Cardamom Tea Bread”, which belongs to the North American cuisine and has the following ingredients: cardamom, egg, vegetable oil, butter, wheat, lemon juice, vanilla, walnut, corn, kumquat, pineapple and tea.

**Figure 4:**
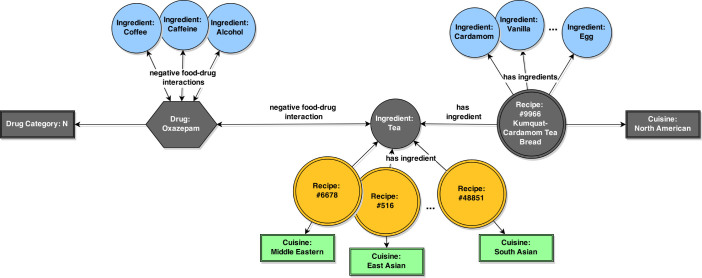
The cuisine - drug interaction concluded from the clinically confirmed negative interaction between Oxazepam and tea. We conclude that Oxazepam, and N drugs in general, have one negative interaction with the North American cuisine, based on this existing connection denoted on the figure with gray elements.

What we do in our analysis is we conclude that in this case tea is responsible for a negative cuisine - drug interaction between the North American cuisine and category N drugs (Fig. 4). We then count this cuisine - drug category interaction as one existing negative interaction.

If this was a case in which recipe #9966, “Kumquat-Cardamom Tea Bread”, contained another ingredient which has a negative interaction with Oxazepam, such as alcohol or coffee, we would still count the interaction between the recipe (and the North American cuisine) and Oxazepam (and the N drug category) as a single negative interaction. We do this because we base our analysis on the ratio between existing (*E_I_*) and possible (*P_I_*) negative interactions, and we calculate the possible negative interactions on a drug - recipe level. Therefore, we have to calculate the number of existing negative interactions on that level, as well.

### Maps

Maps similar to Fig. 2 and Fig. 3 for the other drug categories and ingredients can also be viewed, using our visualization web application (http://viz.linkeddata.finki.ukim.mk/). These maps use data and results from the analysis presented in this paper.

## Author Contributions

M.J., A.B., D.T. and L.K. designed the research. M.J. and A.B. developed tools for the analysis and performed the analysis. M.J., A.B., D.T. and L.K. discussed the results, wrote the paper, and reviewed the manuscript. M.J. and A.B. equally contributed to the work.

## Figures and Tables

**Table 1:** Permils of existing Interactions between Drugs from an ATC Category and Recipes from a Cuisine

	NAm	WEu	NEu	EEu	SEu	MEa	SAs	SEAs	EAs	LAm	Af
A	5.288‰	5.263‰	4.462‰	4.930‰	2.547‰	1.531‰	2.085‰	1.080‰	0.408‰	2.356‰	1.238‰
B	5.271‰	4.345‰	1.643‰	4.687‰	9.663‰	6.894‰	10.640‰	12.699‰	10.756‰	11.013‰	10.045‰
C	5.821‰	5.207‰	3.640‰	5.083‰	6.790‰	4.815‰	7.123‰	8.371‰	6.645‰	8.003‰	6.554‰
D	7.081‰	7.011‰	5.983‰	6.573‰	3.401‰	1.928‰	2.717‰	1.351‰	0.543‰	3.130‰	1.655‰
G	7.240‰	7.504‰	6.326‰	6.836‰	3.789‰	2.848‰	3.520‰	1.781‰	0.528‰	3.237‰	1.652‰
H	0.129‰	0.111‰	0.000‰	0.000‰	0.056‰	0.091‰	0.000‰	0.386‰	0.023‰	0.242‰	0.000‰
J	10.242‰	10.532‰	8.468‰	9.793‰	6.276‰	2.965‰	3.682‰	2.035‰	1.234‰	5.641‰	2.642‰
L	4.430‰	4.673‰	3.818‰	4.156‰	2.744‰	1.644‰	1.720‰	1.276‰	0.844‰	2.137‰	1.442‰
M	0.000‰	0.000‰	0.000‰	0.000‰	0.000‰	0.000‰	0.000‰	0.000‰	0.000‰	0.000‰	0.000‰
N	4.829‰	5.510‰	3.397‰	4.706‰	5.935‰	4.157‰	4.588‰	4.923‰	3.555‰	6.032‰	3.514‰
P	0.078‰	0.067‰	0.000‰	0.000‰	0.034‰	0.055‰	0.000‰	0.234‰	0.014‰	0.147‰	0.000‰
R	0.193‰	0.237‰	0.155‰	0.153‰	0.139‰	0.211‰	0.172‰	0.234‰	0.015‰	0.160‰	0.028‰
S	5.117‰	5.074‰	4.302‰	4.779‰	2.437‰	1.433‰	1.975‰	1.012‰	0.398‰	2.270‰	1.206‰
V	9.022‰	8.023‰	5.123‰	8.150‰	10.912‰	7.453‰	11.498‰	12.822‰	10.888‰	12.582‰	10.815‰

**Table 2:** Percentage of Interactions involving the Ingredient, for all Cuisines

Ingredient	% of Interactions involving the Ingredient
milk	56.110%
garlic	22.617%
coffee	8.388%
ginger	5.109%
cheese	2.197%
bacon	2.165%
red wine	1.865%
grapefruit	1.684%
ham	1.296%
wine	1.174%
tea	1.149%
avocado	0.869%
beer	0.304%
licorice	0.120%

**Table 3:** Top 3 interacting Ingredients per Cuisine

Cuisine	Top 3 interacting Ingredients
North American	milk, garlic, coffee
Western European	milk, garlic, coffee
Northern European	milk, coffee, ginger
Eastern European	milk, garlic, coffee
Southern European	garlic, milk, coffee
Middle Eastern	garlic, milk, coffee
South Asian	garlic, ginger, milk
Southeast Asian	garlic, ginger, milk
East Asian	garlic, ginger, milk
Latin American	garlic, milk, avocado
African	garlic, ginger, milk

**Table 4:** Percentage of Ingredient participation in negative food - drug interactions, per Cuisine

	NAm	WEu	NEu	EEu	SEu	MEa	SAs	SEAs	EAs	LAm	Af
milk	62.288%	60.132%	70.955%	62.030%	32.665%	26.331%	30.302%	13.634%	8.637%	29.196%	23.833%
garlic	17.606%	13.616%	3.119%	17.868%	41.754%	39.943%	45.090%	59.570%	67.848%	47.122%	53.333%
coffee	8.662%	11.213%	10.234%	8.883%	8.711%	14.837%	4.012%	3.671%	0.456%	5.648%	2.917%
ginger	3.929%	2.670%	4.678%	1.218%	0.689%	6.971%	40.352%	32.092%	42.626%	1.399%	28.000%
cheese	1.631%	2.203%	0.780%	2.132%	6.241%	0.754%	0.382%	0.315%	0.182%	7.302%	0.500%
bacon	2.244%	3.344%	0.877%	4.416%	1.795%	0.000%	0.115%	0.000%	0.899%	1.815%	0.250%
grapefruit	1.705%	1.435%	0.000%	0.000%	0.823%	2.025%	0.000%	6.765%	0.560%	3.470%	0.000%
red wine	1.434%	3.337%	3.119%	2.843%	5.303%	5.652%	0.611%	0.210%	1.303%	1.829%	6.000%
ham	1.234%	1.602%	1.462%	0.457%	2.757%	0.000%	0.000%	0.157%	1.133%	0.746%	0.000%
wine	0.847%	1.575%	1.170%	0.203%	2.431%	1.507%	0.611%	4.195%	7.817%	0.403%	1.333%
tea	1.141%	1.589%	3.314%	1.726%	0.000%	4.805%	10.394%	5.349%	0.443%	0.000%	0.000%
avocado	0.554%	0.133%	0.780%	0.000%	0.211%	0.565%	0.000%	0.210%	1.042%	7.746%	0.333%
beer	0.299%	0.601%	0.292%	0.152%	0.101%	0.141%	0.000%	0.000%	0.156%	0.605%	0.000%
licorice	0.141%	0.000%	1.754%	0.000%	0.000%	0.000%	0.000%	0.000%	0.234%	0.000%	0.000%

**Table 5:** Percentage of drugs, per ATC Category, which have negative food interaction with an Ingredient

	A	B	C	D	G	H	J	L	M	N	P	R	S	V
milk	1.923%	0.000%	1.058%	2.609%	2.326%	0.000%	3.509%	1.515%	0.000%	0.431%	0.000%	0.000%	1.887%	1.754%
garlic	0.000%	1.786%	1.058%	0.000%	0.000%	0.000%	0.000%	0.000%	0.000%	0.431%	0.000%	0.000%	0.000%	1.754%
coffee	1.282%	1.786%	1.058%	0.870%	6.977%	0.000%	1.170%	0.758%	0.000%	8.621%	0.000%	0.971%	0.943%	0.000%
ginger	0.000%	1.786%	1.058%	0.000%	0.000%	0.000%	0.000%	0.000%	0.000%	0.431%	0.000%	0.000%	0.000%	1.754%
cheese	0.000%	0.000%	0.000%	0.000%	0.000%	0.000%	0.585%	0.000%	0.000%	0.431%	0.000%	0.000%	0.000%	0.000%
bacon	0.000%	0.000%	0.000%	0.000%	0.000%	0.000%	0.585%	0.000%	0.000%	0.862%	0.000%	0.000%	0.000%	0.000%
red wine	0.000%	0.000%	0.000%	0.000%	0.000%	0.000%	0.585%	0.758%	0.000%	0.862%	0.000%	0.000%	0.000%	0.000%
grapefruit	1.923%	0.000%	9.524%	1.739%	0.000%	5.882%	1.754%	3.030%	0.000%	5.172%	3.571%	1.942%	1.887%	0.000%
ham	0.000%	0.000%	0.000%	0.000%	0.000%	0.000%	0.585%	0.000%	0.000%	0.862%	0.000%	0.000%	0.000%	0.000%
wine^a^	0.000%	0.000%	0.000%	0.000%	0.000%	0.000%	0.585%	0.758%	0.000%	0.862%	0.000%	0.000%	0.000%	0.000%
tea	1.282%	1.786%	1.058%	0.870%	6.977%	0.000%	0.585%	0.758%	0.000%	8.621%	0.000%	0.971%	0.943%	0.000%
avocado	0.000%	0.000%	1.058%	0.000%	0.000%	0.000%	0.000%	0.000%	0.000%	0.862%	0.000%	0.000%	0.000%	1.754%
beer	0.000%	0.000%	0.000%	0.000%	0.000%	0.000%	0.585%	0.000%	0.000%	0.862%	0.000%	0.000%	0.000%	0.000%
licorice	0.000%	0.000%	9.524%	0.000%	0.000%	0.000%	0.000%	0.000%	0.000%	0.000%	0.000%	0.000%	0.000%	0.000%

^a^There is a significant number of recipes which contain ‘wine' as an ingredient. The negative interactions of drugs only contain ‘red wine' explicitly while ‘white wine' is considered safe. Despite the ambiguous nature of the ingredient listed simply as ‘wine' we decided to use it in the analysis since it can still be a cause of negative cuisine - drug interactions in cases when red wine is used in the preparation of the recipe. As we can see from the table the ‘wine' ingredient has negative interactions with the drugs which react negatively with ‘red wine'.

**Table 6:** ATC Code List

Code	Contents
A	Alimentary tract and metabolism
B	Blood and blood forming organs
C	Cardiovascular system
D	Dermatologicals
G	Genito-urinary system and sex hormones
H	Systemic hormonal preparations
J	Antiinfectives for systemic use
L	Antineoplastic and immunomodulating agents
M	Musculo-skeletal system
N	Nervous system
P	Antiparasitic products, insecticides and repellents
R	Respiratory system
S	Sensory organs
V	Various
